# Ethical Approval Waiver in the Shivamogga KFD Outbreak Investigation: Concerns and Call for Expedited Ethics Review Framework

**DOI:** 10.3389/ijph.2025.1608622

**Published:** 2025-07-14

**Authors:** Praveen Kumar S.

**Affiliations:** Department of Community and Family Medicine, All India Institute of Medical Sciences (AIIMS), Mangalagiri, India

**Keywords:** ethical approval, ethical issues, expedited ethics review, outbreak investigation, public health emergencies, research ethics

Dear Editor,

I am writing this letter to express my concerns on the ethical practises in the article titled “Kyasanur Forest Disease: An Epidemiological Investigation and Case-Control Study in Shivamogga, Karnataka, India --2022” by Vedachalam et al., which was published in International Journal of Public Health on 18 October 2024 [1]. The study gives insights on risk factors for Kyasanur Forest Disease (KFD), I find the decision to waive Institutional Ethics Committee (IEC) approval for this research concerning.

The authors justified the waiving of Institutional Ethics Committee (IEC) approval by National Centre for Disease Control (NCDC) citing Epidemic Diseases Act No. 3, 1897, which grants power to the state or central government, to take special measures and prescribe regulations for dangerous epidemic diseases. The study is stated as “public health non-research activity”, addressing urgent health crisis. However, the study generates generalizable knowledge, extending beyond a public health outbreak investigation. The Indian Council of Medical Research (ICMR) ethical guidelines state that research during humanitarian emergencies, including outbreak investigations may have a need to undertake research quickly, “this should not impact scientific validity and the need to uphold ethical requirements” [2]. The World Health Organization (WHO) also state that such studies require IEC review and measures to provide timely ethical approval is to be done by collaboration with local and international bodies, even during outbreaks [3]. So there is no clear explanation for wavering, which raises important ethical questions about transparency and accountability.

However, the authors obtained informed consent and adhered to data protection protocols, still steps alone do not replace the need for independent ethical oversight. An expedited IEC review could have been got to ensure participant protection considering the urgency of the investigation. By waiving IEC approval, it might establish an instance that could weaken ethical standards in future outbreak research, particularly in resource poor settings where oversight of getting IEC approval is already challenging.

To address this, I suggest the journal adopt a policy requiring detailed justification for any IEC waivers in future submissions. Encouraging authors to seek expedited ethical reviews and approval even in emergency contexts. Nationally, clearer guidelines on ethical oversight for outbreak investigations could further ensure that public health research has standards of integrity.

Thank you for considering these concerns. I hope this letter gets a constructive discussion on how we can strengthen ethical practices in public health research while responding effectively to urgent health challenges.

Sincerely,

Dr. Praveen Kumar S.
